# B7-H1 Influences the Accumulation of Virus-Specific Tissue Resident Memory T Cells in the Central Nervous System

**DOI:** 10.3389/fimmu.2017.01532

**Published:** 2017-11-09

**Authors:** Kevin D. Pavelko, Michael P. Bell, Susan M. Harrington, Haidong Dong

**Affiliations:** ^1^Department of Immunology, College of Medicine, Mayo Clinic, Rochester, MN, United States; ^2^Department of Urology, College of Medicine, Mayo Clinic, Rochester, MN, United States

**Keywords:** Theiler’s murine encephalomyelitis virus, B7-H1, programmed death-1, CD8, tissue resident memory, picornaviruses, immune checkpoint, CTL, viruses

## Abstract

Therapies that target the PD-1/B7-H1 axis have revolutionized cancer treatment, yet precise knowledge of how this pathway provides benefit continues to evolve. Here, we report a novel role for the immune checkpoint ligand B7-H1 in the accumulation of tissue-resident memory CD8^+^ T-cells (T_RM_). After intracranial infection, Theiler’s murine encephalomyelitis virus (TMEV) generates T_RM_ that are maintained in the central nervous system (CNS) tissues of B7-H1^WT^ animals. Although no differences in acute T-cell responses between B7-H1^WT^ and B7-H1^KO^ are observed, at long-term periods post-infection the maintenance of CD8^+^ T_RM_ is diminished in B7-H1^KO^ animals. This is accompanied by redistribution of the resident CD8^+^ population from primarily CD103^+^ T_RM_ to a diminished population of T_RM_ and a preponderance of non-specified PD-1^+^ CD103^−^ CD8^+^ T-cells. T-cell transfer studies demonstrate that host B7-H1 is necessary for maintaining T_RM_ and limiting accumulation of PD-1^+^ CD103^−^ CD8^+^ T-cells. The lack of host B7-H1 results in compromised control of a heterologous virus re-challenge demonstrating a functional defect in T_RM_ mediated virus control. This study reveals a new role for B7-H1 in T_RM_ and pro-inflammatory PD-1^+^ CD103^−^ CD8^+^ T-cell accumulation in the CNS and gives insight for using B7-H1/PD-1 blockade in modulating long-term T-cell protection.

## Introduction

The development of immunologic memory is a fundamental process in response to vaccination and is critical for protection from secondary exposure to viral pathogens. However, many questions regarding the establishment and maintenance of immunologic memory remain unanswered. Acute viral infections are often accompanied by the recruitment and expansion of cytotoxic effector CD8^+^ T-cells that target MHC class I restricted viral antigens presented on the surface of infected cells. This targeting promotes killing of the infected cell and clearance of the virus from invaded tissues. Upon viral clearance, the CD8^+^ T-cell population contracts in a regulated process to avoid continuous immune mediated pathology and to restore tissue homeostasis. Although there is a substantial reduction of the effector CD8^+^ T-cell population, the CD8^+^ T-cells that remain persist and become long-term memory CD8^+^ T-cells (T_M_). Upon re-exposure to pathogens, T_M_ respond with increased potency and efficiency and rapidly clear secondary infections. These T_M_ can be classified into subgroups based on their location, cell surface biomarker expression, and transcriptional profiles (1). CD8^+^ central memory cells (T_CM_) and effector memory cells (T_EM_) remain within the lymphoid organs or in circulation and provide a rapid response that is activated upon antigen re-exposure (2). In contrast, tissue resident memory CD8^+^ T-cells (T_RM_) remain at sites of previous infection and provide tissue-specific protection from secondary exposure (3). T_RM_ have been identified in a variety of tissues, including lung, skin, intestine, and brain (4–7). The integrin CD103 has been shown to promote the retention of T_RM_ in brain tissues (7) and interactions with ligands expressed in the brain may provide support for their maintenance and retention within the CNS. Although emphasis continues to be placed on the mechanisms involved in T_CM_ and T_EM_ development in lymphoid organs and circulating blood, more recently interest has grown in understanding tissue-specific T_RM_ development, regulation, and maintenance (8–12).

The expression of the immune checkpoint molecule B7-H1 (PD-L1) and interactions with its receptor programmed death-1 (PD-1) constitute a fundamental mechanism in regulating the magnitude of effector T-cell responses. This response is particularly critical after inflammatory viral infections and activation of CD8^+^ cytotoxic lymphocytes in vital host tissues. The upregulation of PD-1 on T-cells after activation and subsequent interaction with B7-H1 in host tissues ensures that a regulated process of T-cell contraction and elimination of unnecessary inflammation occurs (13). In the presence of chronic antigen exposure, some CD8^+^ T-cells convert to an exhausted state, which coincides with their upregulation of PD-1 and the gradual loss of their ability to eliminate their intended targets (14). These findings have formed the basis for the development of immunotherapeutic interventions aimed at targeting immune checkpoint molecules for the treatment of cancers (15). Some effector CD8^+^ T-cells that ultimately eliminate virus from infected tissues are downregulated yet are maintained as T_RM_ for protection from subsequent re-exposure to pathogens. Although both exhausted CD8^+^ cells and T_RM_ develop after viral infection, it is unclear how specific tissue interactions regulate the development of either of these CD8 T-cell phenotypes. Several factors unique to the responsive T-cell population, the host tissues or the virus have potential roles in the development of these persisting tissue specific T-cell populations.

Intracranial infection with the mouse pathogen Theiler’s murine encephalomyelitis virus (TMEV) is often used as a model of persistent infection and neuropathology in mice expressing susceptible MHC class I alleles (16). However, in mice expressing the H-2D^b^ allele acute encephalitis and virus infection is cleared by an immunodominant CD8^+^ T-cell response to the virus antigen VP2_121–130_ (17). Upon viral clearance, immune-mediated pathology subsides and replicating virus is no longer detected in the central nervous system (CNS). This clearance is preferentially associated with the H-2D^b^ allele in C57BL/6 mice and H-2D^b^ provides exclusive protection in FVB transgenic mice (18). Although acute CD8^+^ T-cell responses to VP2_121–130_ comprise up to 70% of the central nervous system infiltrating lymphocyte (CNS-IL) pool of CD8^+^ T-cells, little is known about the development of brain-specific T_RM_ after infection with this virus. Previously, others have shown a role for immune checkpoint molecule blockade in the protection of the CNS from persistent virus infection and chronic inflammation (19). However, it is unclear what role B7-H1 may play in the generation of T_RM_ after acute encephalitis and viral clearance. To that end, we examined the continued presence of T_RM_ in the CNS at time points greater than 30 days post-TMEV infection using both B7-H1^WT^ and B7-H1^KO^ animals. We found that after the resolution of acute infection, T-cells responsive to the viral peptide VP2_121–130_ and to the embedded peptide antigen OVA_257–264_ developed into T_RM_ and persisted within the CNS for longer than 200 days post-infection in the absence of replicating virus. Further, we identified a role for host B7-H1 in enhancing the accumulation of T_RM_ in the CNS and demonstrated that, as a consequence of that role B7-H1 is required for controlling a secondary virus challenge. These findings have implications for the design and use of immune checkpoint blockade therapies and extend our understanding of the role B7-H1 plays in resolving CNS inflammation and of its contribution to the development of T_RM_.

## Materials and Methods

### Animals and Cell Lines

C57BL/6 (B7-H1^WT^) and C57BL/6 CD45.1 (Ly5.1) mice were purchased from Jackson Laboratories (Bar Harbor, ME, USA). OT-1 TCR (CD90.1^+^) transgenic mice were provided by T. Tian (Harvard University, Boston, MA, USA). B7-H1^KO^ C57BL/6 mice were provided by L. Chen (Yale University, New Haven, CT, USA). All animals were housed in the Mayo Clinic Department of Comparative Medicine and cared for according to the Mayo Clinic Institutional Animal Care and Use Committee and NIH guidelines for animal use and care. All biologic agents were used in accordance with the policies and guidelines designated by the Mayo Clinic Institutional Biosafety Committee.

### Antibodies and Tetramers

The antibodies used for flow cytometry were Brilliant Violet 421™ anti-mouse CD8α (Biolegend; Clone 53-6.7), APC/Cy7 anti-mouse CD8β Antibody (Biolegend; YTS156.7.7), Brilliant Violet 605™ anti-mouse CD45 (Biolegend; 30-F11), PerCP/Cy5.5 anti-mouse CD103 (Biolegend; 2E7), BV421 anti-Mouse CD103 (BD Biosciences; M290), PE-Cyanine7 anti-human/mouse CD44 (Tonbo; IM7), PE anti-mouse CD279/PD-1 (Tonbo; J43.l), FITC anti-mouse CD279/PD-1 (eBioscience; RMP1-30), FITC, anti-mouse CD69 (BD Biosciences; H1.2F3), and FITC anti-mouse CD90.1 (eBioscience; HIS51). Allophycocyanin labeled H-2D^b^/VP2_121–130_, H-2K^b^/SIYR, and H-2K^b^/OVA_257–264_ tetramers were kindly provided by Dr. Aaron Johnson (Mayo Clinic, Rochester, MN, USA) and were generated using previously described methods (17, 20). Anti-PD1 hamster monoclonal antibody (G4) was purified and dosed as previously described (21). Animals were treated with 200 µg of anti-PD-1 or isotype IgG on day three post-infection with TMEV-OVA8. The antibody used for *in vivo* intravascular labeling of CD8^+^ T-cells was PE anti-mouse CD8α (BD Biosciences; 53-6.7). Intravascular labeling of peripheral blood lymphocytes and CNS-IL was performed using previously described methods (22).

### CNS-IL Isolation and FACS Analysis

Isolation of CNS-ILs was performed using previously described methods (23). Briefly, at designated time points, post-infection mice were euthanized with CO_2_ prior to collection of brain and spinal cord into 5 mL of 4°C RPMI. Animals were perfused with 50 mL of PBS prior to tissue harvest to exclude the possibility of contamination by blood-derived rather than tissue-derived cells. Tissues were then transferred to a Pyrex Ten Broeck homogenizer (Corning 7 mL, 0.15 mm gap) and homogenized until complete tissue dissociation is attained (5–7 strokes). The CNS homogenate was then sieved through a Corning™ 100 µm strainer (Fisher Scientific; Cat. No. 08-771-19) followed by addition of 5 mL RPMI. The homogenate was then brought to 70% Percoll prepared in PBS in a final volume of 30 mL prior to centrifugation in at 7,840 *g* for 25 min at 4°C. After centrifugation a top myelin debris layer was removed and isolated cells were resuspended in a total volume of 50 mL RPMI before pelleting at 800 *g*. Red blood cells were lysed with ACK (ammonium chloride potassium) lysing buffer, washed with RPMI, and resuspended in FACS buffer before addition of appropriate fluorochrome labeled antibodies or tetramers and analysis by FACS. Final stained cell populations were spiked with 50 µL of CountBright™ absolute counting beads prior to FACS to determine absolute cell numbers. Cells were run on a BD LSRII flow cytometer (BD Bioscience), and data were analyzed using FloJo Software version 7.6.5 (Tree Star, Ashland, OR, USA). For reinfection experiments mice were depleted of peripheral CD8^+^ cells by injection of 0.5 mg of anti-mouse CD8 antibody (Lyt2.43) on day 7, 5, and 3 prior to reinfection with GD7-KS1 as previously described (24).

### Virus, Infection, and Quantitation

Molecularly defined viruses derived from the two TMEV subgroup viruses were used for these experiments. The XhoI-OVA8 virus is derived from the Daniel’s strain which belongs to the less neurovirulent subgroup TO. The TMEV-GD7-KS1 virus is derived from the GDVII substrain that belongs to the highly neurovirulent subgroup GDVII (25). The XhoI-OVA8 (TMEV-OVA8) and GD7-KS1 (TMEV-GD7) viruses were described previously (26, 27). Mice were infected intraperitoneally with 2 × 10^6^ or intracranially with 2 × 10^5^ PFU of TMEV-OVA8. Virus titers were determined by plaque assay using NCTC clone 929 (L-929) cells (ATCC, Manassas, VA, USA) using a protocol described previously (28). Semi-quantitative RT-PCR targeting the VP2 capsid protein of TMEV-OVA8 was used to determine virus RNA levels and has been described (27).

### *In Vivo* Killing Assay

A modified version of a previously described technique was used to test *in vivo* killing by cytotoxic lymphocyte responses induced with TMEV-OVA8 (29). On day 6 after intraperitoneal infection of B7-H1^WT^ or B7-H1^KO^ mice, three peptide-pulsed target cell populations were prepared from C57BL/6 CD45.1 donor splenocytes. Two concentrations of carboxyfluorescein succinimidyl ester (CFSE; Excitation/Emission 490 nm/520 nm) were used to label the no peptide population (CFSE^Low^) and the virus peptide VP2_121–130_ (FHAGSLLVFM; CFSE^High^). Chicken ovalbumen_257–264_ (SIINFEKL) pulsed splenocytes were labeled with a second dye PKH26 (Ex/Em; 551 nm/567 nm). The three populations were mixed at equal numbers before challenge of TMEV infected mice by intravenous injection. 2 × 10^7^ total cells were injected per mouse. Percent killing was determined by relative number of cells recovered from the splenocytes of infected and naïve animals. Splenocytes were assessed by FACS for the number of cells having the CD45.1 marker and the distribution of the three labeled populations. Percent killing was determined using the following equation: % specific lysis = 1−[*r*_naive_/*r*_infected_] × 100 where: *r* = %CFSE^low^ cells/%CFSE^high^ or PKH26 labeled cells.

### Adoptive Transfer of OT-1 T-Cells and Virus Infection

CD8^+^ T-cells were purified from the spleens of OT-1 (Thy1.1 or Thy1.2) TCR transgenic mice or from OT-1^B7-H1KO^ mice (30) using magnetic bead separation (Miltinyi Biotec, Auburn, CA, USA). After isolation, 2 × 10^6^ CD8^+^ cells were injected intravenously into B7-H1^WT^ or B7-H1^KO^ mice. On the day of transfer mice were infected intracranially with 2 × 10^5^ PFU of TMEV-OVA8. At designated times, post-infection CNS tissues and spleens were harvested and processed for lymphocyte isolation prior to immunophenotyping by FACS.

### Statistics

Mean and SD values were calculated using Excel 2010. Statistical analysis was performed using Sigmaplot for Windows version 11.0. All parametric data were analyzed by *t*-test. Non-parametric data were analyzed by Rank-sum assay. Significance was determined by *P* < 0.05.

## Results

### Intracranial Infection with an Attenuated TMEV Virus Results in the Accumulation and Maintenance of Tissue Resident Memory CD8^+^ T-Cells in the CNS

**Figure 1:**
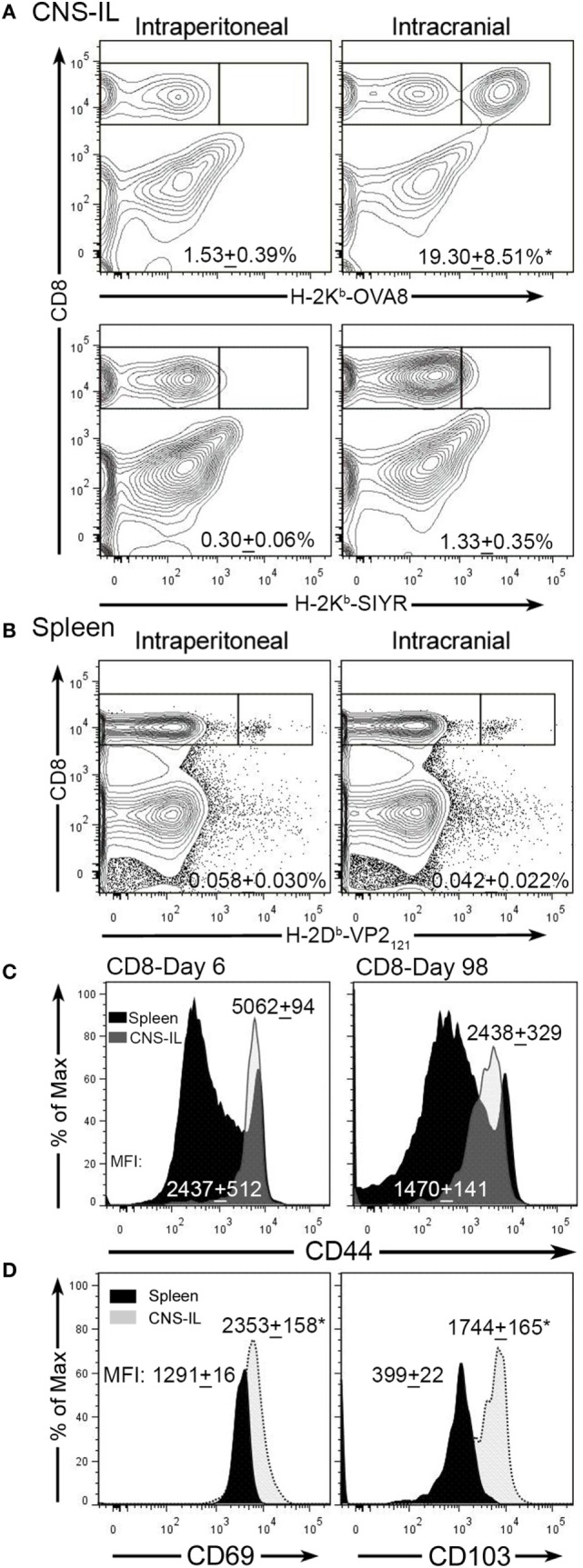
Intracranial infection with Theiler’s murine encephalomyelitis virus (TMEV)-OVA8 generates long lived T_RM_. **(A)** Central nervous system (CNS) infiltrating lymphocytes from intraperitoneally or intracranially infected C57BL/6 mice were analyzed 140 days post-infection for antigen specific CD8^+^ T-cell responses using the virus specific tetramer H-2K^b^-OVA8 or the non-specific control tetramer H-2K^b^-SIYR (*n* = 3 for each group). **(B)** Splenocytes from C57BL/6 infected mice infected with TMEV-OVA8 were analyzed with the virus specific tetramer H-2D^b^-VP2_121–130_ to determine the frequency of virus specific CD8^+^ T-cells. **(C)** Analysis of CD8^+^ cells recovered from the CNS and spleens of C57BL/6 mice for CD44 expression on 6 day 6 and 98 post infection with TMEV-OVA8. Numbers are mean fluorescence intensity (MFI; mean + SD, *n* = 5 per time point). **(D)** CD69 and CD103 expression on H-2K^b^-OVA8^+^ CD8^+^ T-cells isolated from the CNS of C57BL/6 mice intracranially infected with TMEV-OVA8 on day 140 post-infection (*n* = 3 for each group). Data are expressed as mean + SD.

Theiler’s murine encephalomyelitis virus infection of the CNS generates an acute encephalomyelitis that is ultimately cleared by day 14 due to an immunodominant CD8^+^ T-cell response to the viral peptide VP2_121–130_ (18, 31). The generation of this response and the activation of cytotoxic perforin expressing T-cells promotes viral clearance and subsequently inflammation resolves. To determine whether infection with TMEV generates CNS T_RM_, we infected C57BL/6 mice intraperitoneally or intracranially with TMEV-OVA8 and examined the accumulation of virus-specific memory CD8^+^ T-cells 98 days post-infection. This attenuated TMEV virus generates strong CD8^+^ T-cell responses to a virus genome embedded OVA8 peptide as well as the endogenous immunodominant virus antigen VP2_121–130_ (27). At 98 days post-infection, we isolated CNS-ILs and splenocytes from infected animals and found that virus specific H-2K^b^-OVA8^+^ CD8^+^ T-cells (OVA8^+^) were present in the CNS after intracranial infection, but not after intraperitoneal infection (Figure 1A). Recovered splenocytes from both intracranial and intraperitoneal infection generated a population of virus specific OVA8^+^ T-cells, verifying that infection *via* both routes promotes the generation of antigen-specific (VP2^+^) CD8^+^ T cells (Figure 1B). Phenotypic analysis of total CD8^+^ T-cells recovered from the CNS of infected animals revealed that all CD8^+^ T-cells expressed high levels of CD44 (effector/memory T-cell marker) on day 6 but dimmer levels of CD44 at day 98, while CD44 levels were comparable between CD8^+^ T-cells recovered from the spleen (Figure 1C). Further analysis of OVA8^+^ T-cells recovered from both the CNS and spleen demonstrated that the CNS derived virus specific CD8^+^ T-cells expressed high levels of CD69 (T-cell activation marker) and CD103 (tissue-resident memory T-cell marker) compared to spleen derived OVA8^+^ cells (Figure 1D). These findings demonstrate that intracranial TMEV infection results in the development and maintenance of a long lived CNS CD103^+^ CD69^+^ CD8^+^ T_RM_ population.

### B7-H1 Does Not Regulate Acute Cytotoxic T-Cell Function or Alter Virus Clearance

**Figure 2:**
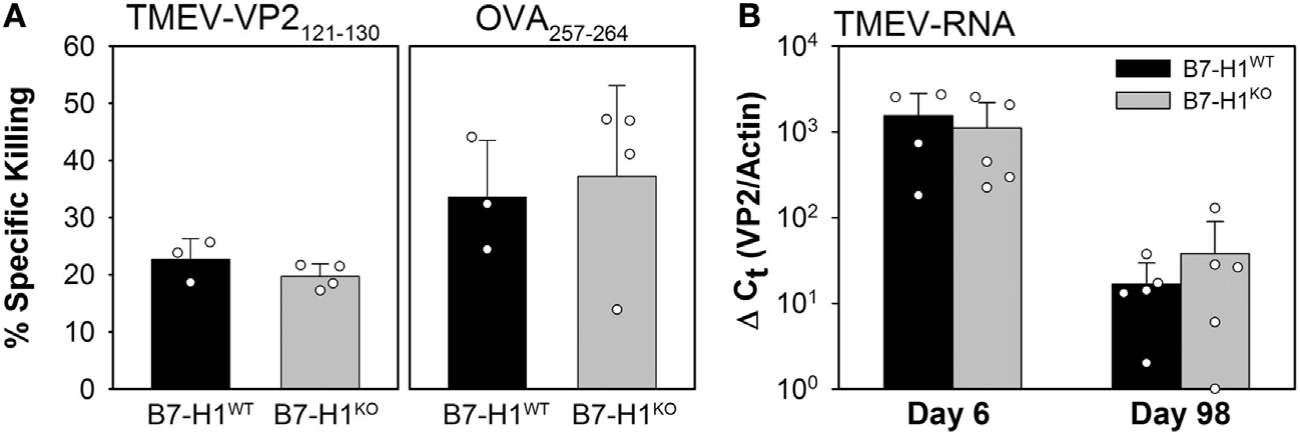
Acute *in vivo* cytotoxic T-cell responses and virus control in the absence of B7-H1. **(A)** *In vivo* killing of the virus-specific peptide [OVA8 and Theiler’s murine encephalomyelitis virus (TMEV)-VP2_121–130_] pulsed target cells recovered from the spleens of B7-H1 competent and B7-H1 deficient mice 6 days post TMEV-OVA8 intraperitoneal challenge. Data are presented as the percent specific killing of virus antigen specific targets recovered and analyzed by FACS relative to unpulsed targets and uninfected control mice. **(B)** RNA purified from the CNS of mice infected intracranially for 6 and 98 days was assessed by semi-quantitative RT-PCR for the presence of TMEV specific transcripts.

To determine the role that B7-H1 plays in the development of virus specific cytotoxic T-cell responses we infected B7-H1 wild-type (B7-H1^WT^) and B7-H1 knockout mice (B7-H1^KO^) with TMEV-OVA8 for 6 days to determine whether immune responses induced by infection can kill virus specific target cells in an *in vivo* CTL assay. We found that the effector T-cells generated by B7-H1^WT^ or B7-H1^KO^ mice equivalently killed both VP2_121–130_ and OVA_257–264_ target cells (Figure 2A). In addition, intracranial infection of B7-H1^WT^ and B7-H1^KO^ mice for 6 or 98 days demonstrated no difference in the level of TMEV RNA obtained from CNS tissues (Figure 2B). A further analysis of CNS homogenates demonstrated that no replicating virus remains in the CNS of C57BL/6 mice after 34 days as assessed by plaque assay, a finding consistent with attenuation of this strain (27) and with previous investigations using intracranial infection with the DA strain of TMEV in C57BL/6 (H-2^b^) mice (32).

### B7-H1 Promotes the Retention of Long-term Virus-Specific T-Cells in the CNS

**Figure 3:**
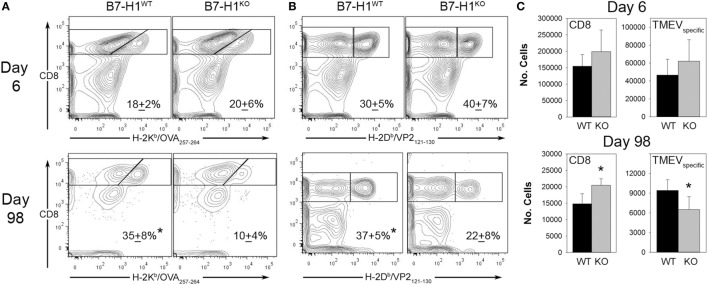
The accumulation of viral antigen specific CD8^+^ T-cells in the CNS of B7-H1^KO^ mice post Theiler’s murine encephalomyelitis virus (TMEV) infection. **(A)** CD45^+^ cells recovered from the CNS were analyzed for percent of total OVA8^+^ CD8^+^ T-cells in B7-H1^WT^ and B7-H1^KO^ mice on day 6 and day 98 post intracranial TMEV-OVA8 infection (*n* = 5 per group; mean + SD percent of total CD8^+^ cells). **(B)** CD45^+^ gated cells in **(A)** were analyzed to determine the percent of B7-H1^WT^ and B7-H1^KO^ CD8^+^ T-cells that were specific to the TMEV peptide antigen VP2_121–130_ on day 6 and day 98. **(C)** Absolute numbers of total CD8^+^ tetramer positive OVA8^+^ and VP2^+^ CD8^+^ T-cells recovered from the CNS of B7-H1^WT^ and B7-H1^KO^ mice on day 6 and day 98 post TMEV-OVA8 intracranial challenge. *denotes significance of *p* < 0.05 by *t*-test when comparing B7-H1^WT^ and B7-H1^KO^.

We next investigated the role of B7-H1 in the development and maintenance of virus specific CD8^+^ T cell responses in the CNS after intracranial infection with TMEV-OVA8. On day 6 post-infection, intracranial infection of either B7-H1^WT^ or B7-H1^KO^ mice revealed no difference in the percentage or absolute numbers of virus specific OVA8^+^ or H-2D^b^-VP2_121–130_^+^ (VP2^+^) cells recovered from the CNS. Although there was a modest increase in total CD8^+^ T-cells recovered from B7-H1^KO^ mice at this acute time point this difference was not significant (*p* = 0.112) (Figures 3A–C).

Long-term post-infection time points were further investigated to determine the role of B7-H1 in promoting the accumulation of virus-specific CD8^+^ T-cells. We found that the quantity of CD8^+^ T-cells remaining in the CNS at 98 days post-infection was approximately 10% of that seen during acute infection in both B7-H1^WT^ and B7-H1^KO^ mice (Figure 3C). To further characterize the residual CD8^+^ T-cell population, we determined the percent and quantity of T-cells that were specific for the known virus specific epitopes. We found that the percentage of OVA8^+^ and VP2^+^ T-cells recovered from the CNS of mice infected for 98 days was decreased in B7-H1^KO^ mice when compared to B7-H1^WT^ mice (Figures 3A,B). In addition, a decrease in the absolute number of virus specific T-cells identified by both tetramers was observed for the CD8^+^ T-cell population in B7-H1^KO^ mice (Figure 3C). These findings suggest that B7-H1 plays a role in the long-term accumulation of T_RM_ cells after intracranial virus infection.

### B7-H1 Influences the Accumulation of PD-1^+^ CD103^−^ CD8^+^ T-Cells in the CNS

**Figure 4:**
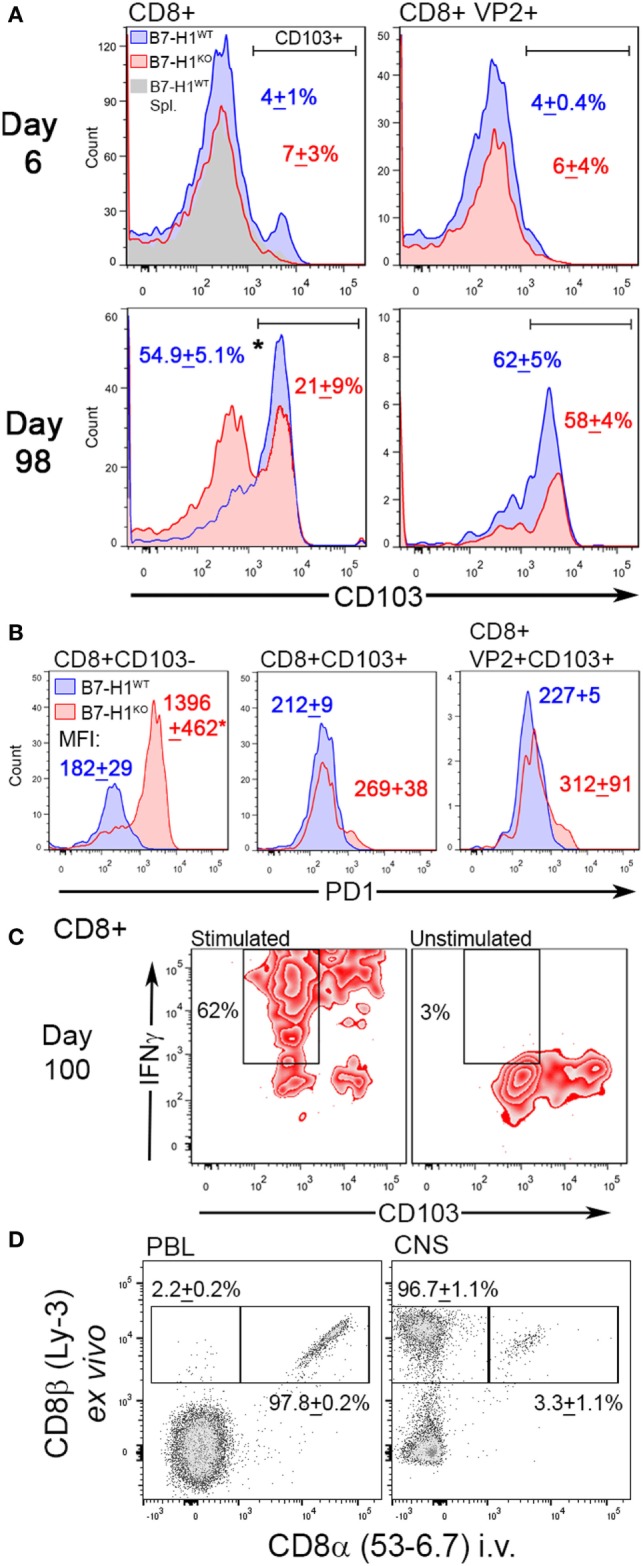
The T_RM_ marker CD103 and the immune checkpoint molecule programmed death-1 (PD-1) distinguish CNS resident CD8^+^ T-cells. **(A)** Expression of CD103 in total CD8^+^ and virus-specific VP2^+^ CD8^+^ CNS-IL recovered from B7-H1^WT^ and B7-H1^KO^ mice on day 6 and 98 days post Theiler’s murine encephalomyelitis virus (TMEV)-OVA8 challenge (*n* = 5 per group; mean percent CD103^+^ SD). Gray histogram—B7-H1^WT^ CD8^+^ splenocyte control. **(B)** PD-1 expression levels in total CD8^+^ and VP2^+^ CD8^+^ CNS-IL recovered from B7-H1^WT^ and B7-H1^KO^ mice on day 99 post-infection. CD103 was used to discriminate T_RM_ and non-T_RM_ populations. (Data are presented as mean fluorescence intensity + SD; *n* = 3.) **(C)** IFNγ secretion relative to CD103 expression in CD8^+^ T-cells recovered from the CNS of B7-H1^KO^ mice infected with TMEV-OVA8 for 100 days. Pooled cells (*n* = 6) were stimulated with PMA/Ionomycin overnight and are compared to unstimulated cells from the same pool. **(D)** Expression of *ex vivo* labeled CD8β and *in vivo* labeled CD8α in B7-H1^KO^ mice infected intracranially with TMEV-OVA8 greater than 90 days prior to analysis. Peripheral blood lymphocytes and CNS-IL were analyzed. (Data are presented as percent CD8β^+^ only and percent CD8β^+^ CD8α^+^ + SD; *n* = 3).

Although our finding suggests that B7-H1 plays a role in promoting CNS-T_RM_ after virus infection, the absolute number of total CD8^+^ T-cells that remain in the CNS of mice infected for 98 days was increased in B7-H1^KO^ mice compared to B7-H1^WT^ (Figure 3C), suggesting that non-specified CD8^+^ T-cell populations fail to contract or are maintained in the CNS at this extended time point post-infection. To determine if phenotypic differences between non-specified and virus specific CD8^+^ T-cells existed, we examined the phenotype of the CNS resident CD8^+^ T-cell populations during acute infection and at 98 days post-infection in B7-H1^WT^ and B7-H1^KO^ mice. First, we assessed the expression levels of CD103 on CD8^+^ T-cells recovered from the CNS of TMEV-OVA8 infected animals, a marker previously used to identify CNS T_RM_ (7, 33). During the acute inflammatory phase of infection, very few of the total CD8^+^ T-cells or the virus-specific VP2^+^ cells expressed the T_RM_ marker CD103, and no differences in this marker were observed between B7-H1^WT^ and B7-H1^KO^ mice (Figure 4A). On day 98 post-infection, a majority of the total CD8^+^ T-cells recovered from the CNS of B7-H1^WT^ animals expressed high levels of CD103. In contrast, this population in B7-H1^KO^ animals was decreased and coincided with an enhanced population of CD8^+^ T-cells that expressed lower levels of CD103 (Figure 4A). Since the phenotypic expression of CD103 in the virus-specific CD8^+^ T-cells was comparable between B7-H1^WT^ and B7-H1^KO^, it seems that the generation of virus-specific T_RM_ was not necessarily influenced by B7-H1 expression. However, the decrease of virus-specific CD8^+^ T-cells in B7-H1^KO^ CNS-IL compared to the CD103^−^ CD8^+^ T-cell population (Figure 3C) suggests that the quality of the virus-specific T_RM_ response and their accumulation in the CNS could be regulated directly by B7-H1 or indirectly by competing cells present in the absence of B7-H1 (Figure 4A).

To investigate potential mechanisms for retention of CD103^−^ CD8^+^ T-cells in the absence of B7-H1, we measured their expression of PD-1, a ligand of B7-H1 identified as a marker for both regulatory T-cells and exhausted T-cells. Of interest, the CD103^−^ CD8^+^ T-cell population that accumulates in the CNS of B7-H1^KO^ mice expressed significantly higher levels of PD-1 compared to that of B7-H1^WT^ mice (Figure 4B). In contrast, total CD103^+^ CD8^+^ T-cells and virus-specific VP2^+^ CD103^+^ T_RM_ derived from the CNS and spleen of B7-H1^WT^ and B7-H1^KO^ mice expressed comparable but low levels of PD-1 (Figure 4B; Figure S1A in Supplementary Material). Since PD-1^high^ CD103^−^ CD8^+^ T-cells dramatically increased in the CNS of B7-H1^KO^ mice, it is possible that their accumulation is directly under the control of B7-H1 when present.

Since CD103^−^ CD8^+^ T-cells represent a population of T-cells that persist in the CNS from early acute infection through time points well beyond virus clearance, we investigated whether CD103^−^ CD8^+^ T-cells were pro-inflammatory cells that persist in the absence of B7-H1. We isolated CNS-IL from B7-H1^KO^ mice and restimulated them *in vitro* to assess the potential for secreting the pro-inflammatory cytokine IFNγ. We found that the non-specific CD103^−^ CD8^+^ T-cells derived from the CNS of B7-H1^KO^ mice secreted IFNγ in response to polyclonal activation of T-cells using PMA/ionomycin (Figure 4C). Similar to a population of CD103^−^ CD8^+^ T-cells present in the spleen of previously infected B7-H1^KO^ mice (Figure S1B in Supplementary Material). Taken together, our findings suggest that B7-H1 promotes the maintenance of PD-1^low^ CD103^+^ T_RM_ by limiting the accumulation of pro-inflammatory PD-1^high^ CD103^−^ CD8^+^ T-cells.

To verify that peripheral blood cells and intravascular lymphocytes do not contribute to the pool of CD8^+^ T-cells that are recovered from the CNS, we performed *in vivo* intravascular staining to discriminate circulating cells from tissue-resident lymphocytes in B7-H1^KO^ mice infected with TMEV-OVA8 greater than 90 days prior to testing. After a 3-min pulse with *in vivo* anti-CD8 antibody, whole peripheral blood and CNS-IL were harvested and analyzed by flow cytometry (Figure 4D). We found that 97.8% of peripheral blood circulating CD8^+^ cells were labeled with the intravascular CD8 antibody, whereas the CNS-IL-derived tissue resident CD8^+^ cells had very few cells labeled by this approach (3.3%). This provides further evidence that the CD8^+^ cells derived by these methods are indeed resident T cells embedded within CNS tissues.

### Host Specific B7-H1 Influences T_RM_ Maintenance in the CNS

**Figure 5:**
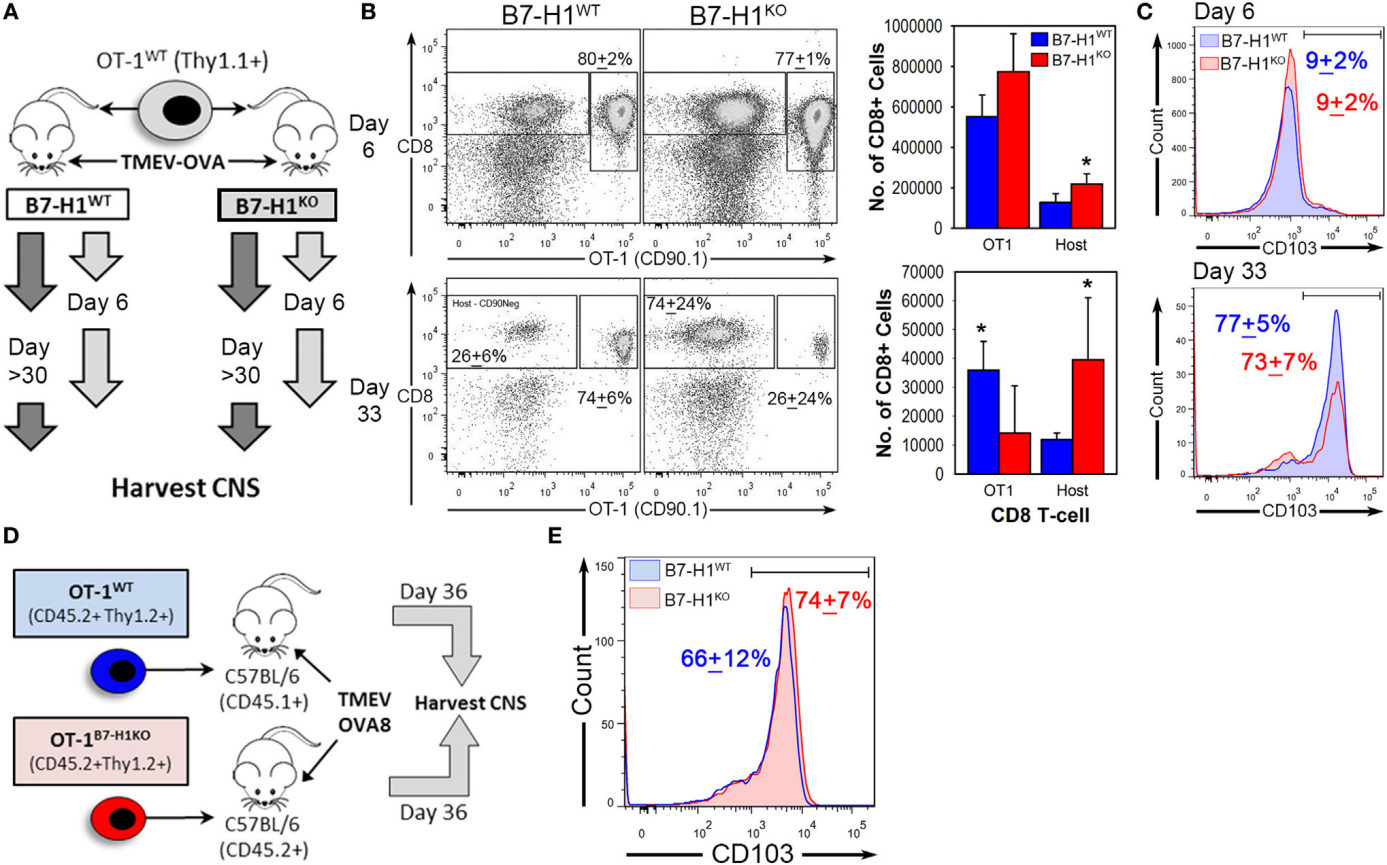
Host-specific B7-H1 promotes central nervous system (CNS) retention of transferred virus specific T_RM_ after intracranial virus infection. **(A)** Adoptive transfer strategy to determine the consequences of B7-H1 expression on T-cells versus host tissues. B7-H1^WT^ CD90.1^+^ OT-1 T-cells were transferred into B7-H1^WT^ CD90.2^+^ hosts (C57BL/6) or into B7-H1^KO^ CD90.2^+^ hosts before intracranial infection with Theiler’s murine encephalomyelitis virus (TMEV)-OVA8. Tissues were harvested on day 6 and day 33 post-infection. **(B)** Total CD45^+^ cells were analyzed for frequency and absolute numbers of recovered CD90.1^+^ CD8^+^ OT-1 and host CD8^+^ T-cells (Host) on day 6 and 33 post TMEV-OVA8 infection from the CNS infected animals. (Percent of total recovered CD90.1^+^ CD8^+^ T-cells + SD.) **(C)** CD103 expression of recovered CD90.1^+^ OT-1 T-cells recovered from the CNS of B7-H1^WT^ and B7-H1^KO^ mice on day 6 and day 33 post intracranial infection with TMEV-OVA8. **(D)** Adoptive transfer strategy and TMEV-OVA8 infection of C57BL/6 recipient hosts of B7-H1^WT^ and B7-H1^KO^ CD8^+^ OT-1 T-cells. **(E)** CD103 expression of B7-H1WT and B7-H1KO CD8^+^ OT-1 T-cells recovered from the CNS of C57BL/6 hosts on day 36 post intracranial infection. *Significant by *t*-test *p* < 0.05; B7-H1^WT^ versus B7-H1^KO^.

Previously, we have shown that expression of B7-H1 on tissue or on T-cells can modulate the function of CD8^+^ T-cells (13, 34). To determine the role of host tissue-specific B7-H1 expression on the regulation of CD8^+^ T_RM_, we transferred congenically marked wild-type OT-1 T-cells into B7-H1^WT^ or B7-H1^KO^ host animals prior to infection with TMEV-OVA8 (Figure 5A). After 6 days of infection, the percent and number of transferred OT-1 T-cells that infiltrated the CNS were comparable between B7-H1^WT^ and B7-H1^KO^ host mice (Figures 5B,C). In addition, the endogenous virus-specific T_RM_ cell percentages were comparable to those observed previously in B7-H1^WT^ and B7-H1^KO^ animals without adoptive transfer (Figures S2A,B in Supplementary Material). On day 33 post-infection, the percentage and number of recovered OT-1 CD8^+^ T-cells were lower in the CNS of B7-H1^KO^ mice compared to B7-H1^WT^ mice (Figure 5B). To verify the conversion of transferred OT-1 T-cells to T_RM_, we measured the levels of CD103 expression on OT-1 T-cells on day 6 and 33 post-infection. We found that the majority of OT-1 T-cells shifted from CD103^−^ at day 6 to CD103^+^ at day 33 (Figure 5C). In addition, the absolute number of CD103^+^ OT-1 T_RM_ cells decreased in the absence of B7-H1, from 27,982 + 8,723 cells (mean + SD) in B7-H1^WT^ to 9,602 + 10,647 cells in B7-H1^KO^ (*t*-test, *p* = 0.017). Endogenous host CD8^+^ T-cells increased in B7-H1^KO^ mice compared to B7-H1^WT^ (Figure 5B) which was due to an increased number of non-specified CD103^−^ CD8^+^ T-cells (Figures S2A,B in Supplementary Material). These results suggest that host-specific B7-H1 influences the accumulation of CD103^+^ T_RM_ in the CNS post-TMEV infection.

To determine whether T-cell specific B7-H1 expression would affect the accumulation of T_RM_, we transferred OT-1 wild-type T-cells (OT-1^WT^) or B7-H1 deficient OT-1 CD8^+^ T-cells (OT-1^B7-H1KO^) into wild-type mice before intracranial infection with TMEV-OVA8 (Figure 5D). The frequencies of OT-1^WT^ and OT-1^B7-H1KO^ T_RM_ were comparable on day 36 post-infection (Figure 5E), demonstrating that B7-H1 expressed by the CD8^+^ OT-1 T-cells does not contribute to the accumulation of T_RM_ in the CNS. Together, our data suggest that host specific B7-H1, but not T-cell B7-H1 promotes accumulation of CD103^+^ T_RM_ in the CNS.

### B7-H1 Is Required for Virus Control upon Secondary Brain Infection

**Figure 6:**
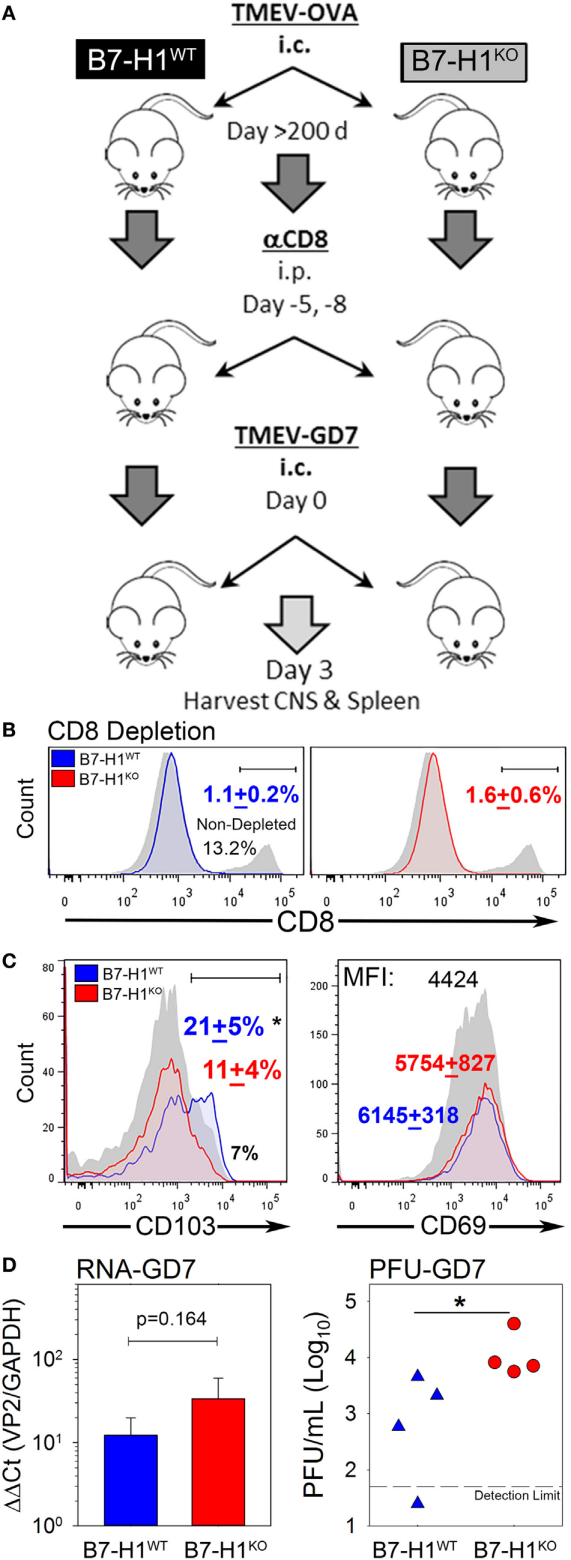
B7-H1 promotes tissue resident memory CD8^+^ T-cell protection from heterologous secondary infection. **(A)** Virus re-challenge scheme to determine the role of B7-H1 in protecting mice from a secondary tissue-specific re-challenge with the Theiler’s murine encephalomyelitis virus (TMEV) variant GD7. **(B)** Splenocytes were analyzed for CD8 expression in B7-H1WT and B7-H1KO mice depleted with CD8 depleting antibody for verify depletion of peripheral CD8^+^ T-cells. Splenocytes were gated on forward-side scatter and CD45 subsequent to analysis of the percent of CD8^+^ cells (*n* = 4 per group). One non-CD8 depleted animal infected with TMEV-OVA8 is used as a reference to determine relative depletion (gray/black). **(C)** CD103 and CD69 expression on CD8^+^ T-cells recovered from the CNS on day 3 after TMEV-GD7 re-challenge. **(D)** Semi-quantitative RT-PCR analysis for TMEV-GD7 specific transcripts from the CNS of B7-H1^WT^ and B7-H1^KO^ mice given a re-challenge with TMEV-GD7. (Right) Plaque assay performed on CNS homogenates recovered from re-infected B7-H1^WT^ and B7-H1^KO^ mice (PFU, plaque-forming units). *Significant by *t*-test *p* < 0.05; B7-H1^WT^ versus B7-H1^KO^.

Since B7-H1 promotes the maintenance of T_RM_, we asked whether lack of B7-H1 would affect virus clearance in the CNS after secondary infection. To address this question, we used the highly pathogenic TMEV variant GD7-KS1 (TMEV-GD7) as a heterologous secondary CNS infection (26), this TMEV variant contains genetic determinants known to increase neuropathology and is attributed to high pathogenicity and lethality even at low doses when injected directly into the CNS (25). Although few cross neutralizing antibody epitopes have been defined, the neutralization profiles are unique (35). Importantly, the immunodominant CD8 T-cell epitope VP2_121–130_ is conserved between TMEV-OVA8 (primary infection) and TMEV-GD7 (secondary infection), allowing direct assessment of virus specific T_RM_ function in the CNS. We first infected B7-H1^WT^ and B7-H1^KO^ mice with TMEV-OVA8. Then to evaluate the potential protection from T_RM_ on day 277 post-infection, we eliminated circulating CD8^+^ T-cells using an anti-CD8 depleting antibody. After CD8 T-cell depletion, the previously infected mice were re-infected intracranially with TMEV-GD7 (Figure 6A). Three days post-challenge CNS-IL and splenocytes were isolated from rechallenged mice to verify peripheral CD8 depletion and to determine the phenotype of CNS derived cells. 8 days after the final dose of CD8 depleting antibody and 3 days after virus rechallenge CD8 depletion was maintained with a 91.4 + 1.3% reduction in total CD8^+^ T-cells in the spleen of B7-H1^WT^ mice and an 87 + 4.2% reduction in B7-H1^KO^ mice compared to non-depleted control (Figure 6B). CNS-IL were analyzed by flow cytometry to determine the phenotype of resident CD8^+^ T-cells. Consistent with previous experiments, this cohort of B7-H1^KO^ mice had a reduced number of CD8^+^ T_RM_ compared to the B7-H1^WT^ group (Figure 6C).

Further, TMEV load was assessed from the CNS of B7-H1^WT^ and B7-H1^KO^ animals by analysis of viral RNA and replicating virus. The levels of TMEV RNA were comparable between the CNS of B7-H1^KO^ and B7-H1^WT^ mice (*p* = 0.164), suggesting viral RNA was present as either encapsidated functional virions or as residual viral transcripts present after virus inactivation. However, more plaque forming units were identified in the CNS of B7-H1^KO^ mice compared to B7-H1^WT^ mice (Figure 6D), suggesting that host B7-H1 is required to promote protection from secondary CNS virus infection by promoting the accumulation of virus-specific CD8^+^ T_RM_ in the CNS.

### Systemic Delivery of PD-1 Blockade during Acute Virus Infection Fails to Modulate CNS T_RM_

**Figure 7:**
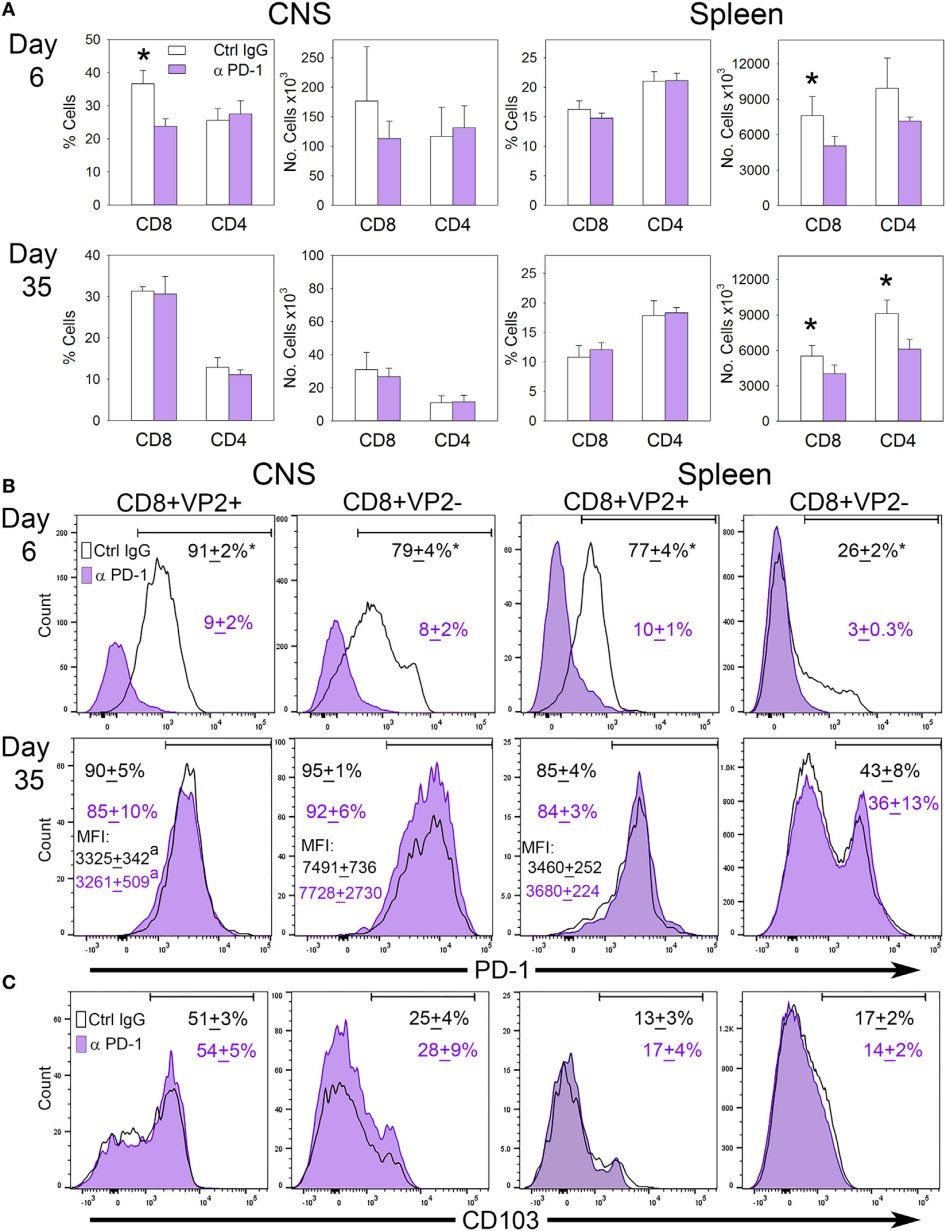
The influence of programmed death-1 (PD-1) blockade on the development of virus specific T_RM_ in B7-H1^KO^ mice after CNS infection with Theiler’s murine encephalomyelitis virus (TMEV)-OVA8. **(A)** Percent and number of CD8^+^ and CD4^+^ cells recovered from the CNS and spleen from B7-H1^KO^ mice infected intracranially and then treated with control poly IgG antibody (black outline) or anti-PD-1 (G4; purple) on day 3 post-infection. CNS infiltrating lymphocytes and spleen cells were analyzed by flow cytometry on day 6 (Ctrl. IgG *n* = 4, anti-PD-1 *n* = 5) and day 35 (Ctrl. IgG *n* = 5, anti-PD-1 *n* = 5) post-infection. **(B)** CD8^+^ cells in **(A)** assessed for VP2^+^ specificity and PD-1 expression and **(C)** the TRM marker CD103. Significant by *t*-test *p* < 0.05 *Control IgG versus anti-PD-1 or CD8^+^ VP2^+^ versus CD8^+^ VP2^−^.

Since B7-H1 influences the accumulation of PD-1^+^ CD103^−^ CD8^+^ T-cells in the CNS, we hypothesized that targeting PD-1 on these cells may potentially restore the T_RM_ population in the CNS by eliminating their presence in the CNS. To address this, we infected B7-H1^KO^ mice intracranially with TMEV-OVA8 and treated mice with control IgG or anti-PD-1 antibody on day 3 post-infection and assessed the distribution of CD8^+^ and CD4^+^ T-cells in the CNS and spleen. At 6 days and 35 days post-challenge, CD45^+^ CNS-IL and spleen cells were analyzed by flow cytometry. On day 6 post-infection, the percentage of CD8^+^ CNS-IL and the absolute number of CD8^+^ cells in the spleen was decreased in the PD-1 blockade group (Figure 7A). By day 35; however, no difference in the percentage or absolute numbers of CD8^+^ or CD4^+^ cells were detected in the CNS of B7-H1^KO^ mice when comparing anti-PD-1-treated versus control IgG-treated animals. Decreases in the absolute numbers of CD8^+^ and CD4^+^ cells were observed in the spleen at day 35 in groups treated with anti-PD-1 compared to control IgG-treated group (Figure 7A).

To further interrogate the CD8^+^ memory populations, we analyzed CD8^+^ VP2^+^ cells by flow cytometry to determine how PD-1 blockade influences the accumulation of virus-specific and non-specific CD8^+^ PD-1^+^ cells in the CNS and spleen. We found that the populations of PD-1^+^ cells in the CD8^+^ VP2^+^ and the CD8^+^ VP2^−^ subsets were significantly reduced using anti-PD-1 antibody demonstrating that PD-1 blockade reduces the CD8^+^ PD-1^+^ population in both the CNS and the spleen at the 6 day acute time point. After 35 days, the differences between the anti-PD-1 and control IgG groups resolve in the CNS and spleen (Figure 7B). Furthermore, the expression of the T_RM_ marker CD103 is consistent with the retention of a CD103^−^ population of cells in the CNS of B7-H1^KO^ animals that is not influenced by systemic treatment with PD-1 blockade (Figure 7C). Of interest, the expression levels of PD-1 in the VP2^−^ populations in both treatment groups at day 35 were increased in relation to the VP2^+^ group suggesting that the accumulation of the CD8^+^ CD103^−^ PD-1^high^ population in the CNS was not influenced by PD-1 blockade (Figures 7B,C). We hypothesize that the inability of the anti-PD-1 antibody to access the CNS through the blood–brain barrier may prevent therapeutic targeting of PD-1 expressing cells in the CNS.

## Discussion

Although much attention has been focused on the generation of tissue resident memory T-cells (T_RM_) in protection from infection or cancer, less is known about how these cells accumulate in distinct niches throughout the body. In this study, we identified a novel role for the immune checkpoint ligand B7-H1 in the promotion of T_RM_ accumulation in the CNS after intracranial viral infection with TMEV. We find that B7-H1 expression in host tissues is important for restricting the inflammatory PD-1^high^ CD103^−^ CD8^+^ T-cells that limit T_RM_ in the CNS. We found a deficit in the quality and quantity of virus-specific T_RM_ in the CNS of B7-H1 deficient hosts after intracranial infection with TMEV, a virus that is ultimately cleared by CD8^+^ T-cell responses to virus antigens. Similarly, others have reported a reduction of T_RM_ in the brain of B7-H1^KO^ mice following infection with the herpes virus murine cytomegalovirus (MCMV) (36). Our findings suggest a new role for B7-H1 within the CNS which includes promotion of T_RM_ maintenance through restriction of PD-1^high^ CD103^−^ CD8^+^ T-cells that accumulate in the absence of B7-H1.

The interaction of cell surface PD-1 expressed by CD8^+^ T-cells with the PD-1 ligand B7-H1 is important for resolving CD8^+^ T-cell responses and preventing sustained immunopathology. One mechanism by which T cell responses are resolved is through the induction of T-cell apoptosis induced by PD-1 ligation in tissues that express B7-H1 (13). Consistent with this hypothesis, we find that B7-H1 may be involved in the depletion of non-specified inflammatory PD-1^high^ CD8^+^ T-cells in the CNS following viral infection. One hallmark of these T-cells is high expression of PD-1, suggesting that in the absence of ligand B7-H1, inflammatory CD8^+^ T-cells with high levels of PD-1 would compete for long-term residence with virus-specific T_RM_ in the CNS post-infection. Importantly, these PD-1^high^ CD8^+^ T-cells may regulate the accumulation of virus-specific T_RM_. One potential hypothesis is that B7-H1 limits the accumulation and survival of low-affinity or antigen non-specific CD8^+^ T-cells following clearance of infections in order to provide space for tissue resident T-cells that are protective against secondary infections, a finding consistent with an associated regulatory function of PD-1 expressing cells (37).

We found that the increased population of CD103^−^ CD8^+^ T-cells observed in B7-H1^KO^ mice produced IFNγ upon stimulation. It is plausible that IFNγ produced by CD103^−^ pro-inflammatory T-cells suppressed the accumulation of T_RM_ in B7-H1^KO^ mice. The role of IFNγ in suppression of memory CD8^+^ T-cells has been reported in lung following influenza infection (38). Although IFNγ does not directly inhibit T-cell proliferation, it suppresses memory T-cell generation through two processes, including the induction of activation-induced cell death of effector cells (39, 40) and through activation of T-bet and its subsequent inhibition of IL-7 receptor expression (41). A consequence of IL-7 receptor signaling in T-cells is reduced levels of bcl-2, a pro-survival molecule needed for memory T-cell survival. Further, low expression of bcl-2 was observed in CD103^+^ T_RM_ cells in the brain of B7-H1^KO^ mice following MCMV infection (36). Together, these studies suggest that IFNγ produced by PD-1^high^ CD103^−^ pro-inflammatory T-cells may contribute to the decreased number of T_RM_ in the CNS of B7-H1^KO^ mice. Future studies are warranted to determine how this unique population of PD-1^high^ inflammatory CD8^+^ T-cells modulates virus antigen-specific T_RM_.

Previous studies have demonstrated that intracranial virus infection results in the accumulation and maintenance of a persistent population of activated CD8^+^ T-cells that persist in the absence of continued virus replication (42). Bergmann et al. found that both antigen specific and non-specific cells accumulate in the CNS after virus clearance with limited cytolytic and pro-inflammatory activity (43). Similarly, we identified two unique populations of CNS antigen-specific and -non-specific CD8^+^ T-cells that can be discriminated by their expression of PD-1. We find that both inflammatory non-specific PD-1^high^ CD8 T-cells and PD-1^low^ antigen-specific T_RM_ were sensitive to the presence of B7-H1. Further, we found that the T_RM_ within B7-H1^WT^ or B7-H1^KO^ CNS tissues express a low level of PD-1 compared to inflammatory PD-1^high^ CD103^−^ CD8^+^ T-cells. This finding suggests that the low level PD-1 expression may prevent T_RM_ in the CNS from a strong engagement with B7-H1 that would lead to T-cell apoptosis or exhaustion. It is not clear why PD-1 levels are different in these two T-cell subsets, but cues within the tissue microenvironment along with TCR signals may differentially regulate PD-1 expression (44, 45). It should be noted that stimulation through a strong T-cell receptor interaction may not be the only signal promoting PD-1 expression, since antigen-specific T_RM_ express low levels of PD-1, while antigen-non-specific CD103^−^ CD8^+^ T-cells expressed higher levels of PD-1 compared to T_RM_ after viral infection.

Although PD-1 has been proposed as a biomarker for exhausted CD8^+^ T-cells that develop during chronic viral infections (14, 46, 47), PD-1 expression *per se* does not always lead to T cell exhaustion and its absence is not necessary for the prevention of exhaustion (48). Since the inflammatory PD-1^high^ CD103^−^ CD8^+^ T-cells can persist in the CNS for extended periods post-infection in the absence of B7-H1, suggesting that the expression of PD-1, even at high levels, did not affect their residence in the CNS. A possible scenario following viral infection in the CNS is that host B7-H1 eliminates inflammatory PD-1^high^ CD103^−^ CD8^+^ T-cells in order to preserve the virus specific T_RM_ that are necessary for protection from secondary infection. It is also possible that the PD-1^high^ inflammatory CD8^+^ T cells represent a new subset of regulatory CD8^+^ T-cells that increases in B7-H1 deficient environments (49). The similarities observed between the inflammatory CD8^+^ T-cells here and regulatory CD8^+^ T-cells warrants further investigation.

The development of immunity in vital organs must be carefully regulated to provide sufficient control from long lasting pathology and protection of tissues with limited renewal capacity. The regulation of B7-H1 expression in the brain and how it can affect the long-term accumulation of CD8^+^ T-cells has implications for studies of immune-mediated neurologic disease. Although baseline B7-H1 expression levels are thought to be low in the CNS, it is often upregulated after injury or infection (50–52). Our study demonstrates that the level of B7-H1 expression in host tissues can determine the quality of the CD8^+^ T-cell population that occupies the CNS. Previous evidence demonstrates that brain resident CD8^+^ T-cells accumulate with age (53). However, it is unclear under what consequences these CD8^+^ T-cells reach the brain and accumulate. One could speculate that over a lifetime, clinical or subclinical CNS infections and their associated inflammatory cascades could lead to an accumulation of CD8^+^ T_RM_ in the brain and spinal cord. However, aging is also associated with deterioration of the blood brain barrier and an increase in immunosurvellance that allows CD8^+^ T-cells to enter the CNS under non-inflammatory conditions (54). Nevertheless, both inflammatory and non-inflammatory mechanisms can promote the accumulation of T_RM_. Our findings here suggest that the level of B7-H1 expressed by host tissues can have consequences for the quality and quantity of the CD8^+^ T-cell subsets that accumulate within CNS tissues.

The discovery of immune checkpoints and the use of reagents that target these pathways have revolutionized cancer immunotherapy. However, their development and use in clinical trials for chronic viral infections or for long-term tumor control has been met with mixed success (55, 56). Similarly here we find that PD-1antibody fails to deplete or influence the PD-1^high^ expressing CNS resident population of CD8^+^ cells or to enhance T_RM_ accumulation. The major difference between targeting B7-H1/PD-1 pathway in cancer and in viral infection is the cellular sources of B7-H1 or PD-1. In the tumor tissues, the blockade of PD-1/B7-H1 is mainly aimed at blocking the interaction between PD-1^+^ effector T cells and B7-H1^+^ tumor cells. However, in the infected tissues, since both effector and regulatory cells express PD-1, the net outcome of the blockade of PD-1/B7-H1 would be determined by a balance of the functions of effector cells and immune regulatory cells. To that end, our study identified a unique population of PD-1^high^ CD103^−^ CD8^+^ T-cells as a potential new cellular target of B7-H1/PD-1 therapy in chronic viral infections. However, since unique barriers exist in protected tissues like the CNS, the use of antibody based therapies will be challenging (57). A prolonged blockade of B7-H1 or PD-1 may provide a risk for the accumulation of the inflammatory PD-1^high^ CD8^+^ T cells that eventually would impair the maintenance of protective T_RM_ in particular tissues. To reduce the possibility of compromising long-term protection mediated by tissue resident CD8^+^ T cells, the timing and location of B7-H1/PD-1 blockade as a therapy for virus vaccines or infection should be carefully optimized in order to support the generation and maintenance of a pool of antigen specific CD8^+^ T_RM_.

In summary, our study reveals a new function of B7-H1 for the promotion of T_RM_ maintenance by limiting the accumulation of inflammatory PD-1^high^ CD103^−^ CD8^+^ T cells in the CNS. This finding extends our understanding of the maintenance of T_RM_ in the brain and provides new insights for optimizing B7-H1/PD-1 blockade to promote long-term protection from cancer or virus infection.

## Ethics Statement

This study was carried out in accordance with the recommendations of the National Institute of Health and the Mayo Clinic Department of Comparative Medicine. The protocol was approved by the Mayo Clinic Institutional Animal Care and Use Committee.

## Author Note

One Sentence Summary: The immune checkpoint molecule B7-H1 modulates the accumulation of tissue resident CD8 T-cells.

## Author Contributions

HD and KP conceived the project and were responsible for research design, data analysis, and drafting the manuscript. KP and MB performed the experiments and data acquisition. SH helped with the animal models.

## Conflict of Interest Statement

The authors declare that the research was conducted in the absence of any commercial or financial relationships that could be construed as a potential conflict of interest.

## References

[1] Mueller SN Gebhardt T Carbone FR Heath WR. Memory T cell subsets, migration patterns, and tissue residence. Annu Rev Immunol (2013) 31:137–61. 10.1146/annurev-immunol-032712-095954 23215646

[2] Sallusto F Lenig D Forster R Lipp M Lanzavecchia A. Two subsets of memory T lymphocytes with distinct homing potentials and effector functions. Nature (1999) 401:708–12. 10.1038/44385 10537110

[3] Masopust D Vezys V Marzo AL Lefrancois L. Preferential localization of effector memory cells in nonlymphoid tissue. Science (2001) 291:2413–7. 10.1126/science.1058867 11264538

[4] Gebhardt T Wakim LM Eidsmo L Reading PC Heath WR Carbone FR. Memory T cells in nonlymphoid tissue that provide enhanced local immunity during infection with herpes simplex virus. Nat Immunol (2009) 10:524–30. 10.1038/ni.1718 19305395

[5] Masopust D Choo D Vezys V Wherry EJ Duraiswamy J Akondy R Dynamic T cell migration program provides resident memory within intestinal epithelium. J Exp Med (2010) 207:553–64. 10.1084/jem.20090858 20156972 PMC2839151

[6] Teijaro JR Turner D Pham Q Wherry EJ Lefrancois L Farber DL. Cutting edge: tissue-retentive lung memory CD4 T cells mediate optimal protection to respiratory virus infection. J Immunol (2011) 187:5510–4. 10.4049/jimmunol.1102243 22058417 PMC3221837

[7] Wakim LM Woodward-Davis A Bevan MJ. Memory T cells persisting within the brain after local infection show functional adaptations to their tissue of residence. Proc Natl Acad Sci U S A (2010) 107:17872–9. 10.1073/pnas.1010201107 20923878 PMC2964240

[8] Iijima N Iwasaki A. Tissue instruction for migration and retention of TRM cells. Trends Immunol (2015) 36:556–64. 10.1016/j.it.2015.07.002 26282885 PMC4567393

[9] Carbone FR. Tissue-resident memory T cells and fixed immune surveillance in nonlymphoid organs. J Immunol (2015) 195:17–22. 10.4049/jimmunol.1500515 26092813

[10] Korn T Kallies A T cell responses in the central nervous system. Nat Rev Immunol (2017) 17(3):179–94. 10.1038/nri.2016.144 28138136

[11] Bergsbaken T Bevan MJ Proinflammatory microenvironments within the intestine regulate the differentiation of tissue-resident CD8(+) T cells responding to infection. Nat Immunol (2015) 16:406–14. 10.1038/ni.3108 25706747 PMC4368475

[12] Schenkel JM Fraser KA Casey KA Beura LK Pauken KE Vezys V IL-15-independent maintenance of tissue-resident and boosted effector memory CD8 T cells. J Immunol (2016) 196:3920–6. 10.4049/jimmunol.1502337 27001957 PMC5145194

[13] Dong H Zhu G Tamada K Flies DB van Deursen JM Chen L. B7-H1 determines accumulation and deletion of intrahepatic CD8(+) T lymphocytes. Immunity (2004) 20:327–36. 10.1016/S1074-7613(04)00050-0 15030776

[14] Day CL Kaufmann DE Kiepiela P Brown JA Moodley ES Reddy S PD-1 expression on HIV-specific T cells is associated with T-cell exhaustion and disease progression. Nature (2006) 443:350–4. 10.1038/nature05115 16921384

[15] Melero I Hervas-Stubbs S Glennie M Pardoll DM Chen L. Immunostimulatory monoclonal antibodies for cancer therapy. Nat Rev Cancer (2007) 7:95–106. 10.1038/nrc2051 17251916

[16] Rodriguez M Leibowitz J David CS. Susceptibility to Theiler’s virus-induced demyelination. Mapping of the gene within the H-2D region. J Exp Med (1986) 163:620–31. 10.1084/jem.163.3.620 3005466 PMC2188055

[17] Johnson AJ Njenga MK Hansen MJ Kuhns ST Chen L Rodriguez M Prevalent class I-restricted T-cell response to the Theiler’s virus epitope Db:VP2121-130 in the absence of endogenous CD4 help, tumor necrosis factor alpha, gamma interferon, perforin, or costimulation through CD28. J Virol (1999) 73:3702–8. 10196262 10.1128/jvi.73.5.3702-3708.1999 PMC104145

[18] Mendez-Fernandez YV Johnson AJ Rodriguez M Pease LR. Clearance of Theiler’s virus infection depends on the ability to generate a CD8+ T cell response against a single immunodominant viral peptide. Eur J Immunol (2003) 33:2501–10. 10.1002/eji.200324007 12938226

[19] Duncan DS Miller SD. CNS expression of B7-H1 regulates pro-inflammatory cytokine production and alters severity of Theiler’s virus-induced demyelinating disease. PLoS One (2011) 6:e18548. 10.1371/journal.pone.0018548 21494618 PMC3072984

[20] Altman JD Moss PA Goulder PJ Barouch DH McHeyzer-Williams MG Bell JI Phenotypic analysis of antigen-specific T lymphocytes. Science (1996) 274:94–6. 10.1126/science.274.5284.94 21690331

[21] Hirano F Kaneko K Tamura H Dong H Wang S Ichikawa M Blockade of B7-H1 and PD-1 by monoclonal antibodies potentiates cancer therapeutic immunity. Cancer Res (2005) 65:1089–96. 15705911

[22] Anderson KG Mayer-Barber K Sung H Beura L James BR Taylor JJ Intravascular staining for discrimination of vascular and tissue leukocytes. Nat Protoc (2014) 9:209–22. 10.1038/nprot.2014.005 24385150 PMC4428344

[23] Cumba Garcia LM Huseby Kelcher AM Malo CS Johnson AJ. Superior isolation of antigen-specific brain infiltrating T cells using manual homogenization technique. J Immunol Methods (2016) 439:23–8. 10.1016/j.jim.2016.09.002 27623324 PMC5310589

[24] Steinbach K Vincenti I Kreutzfeldt M Page N Muschaweckh A Wagner I Brain-resident memory T cells represent an autonomous cytotoxic barrier to viral infection. J Exp Med (2016) 213:1571–87. 10.1084/jem.20151916 27377586 PMC4986533

[25] Fu JL Stein S Rosenstein L Bodwell T Routbort M Semler BL Neurovirulence determinants of genetically engineered Theiler viruses. Proc Natl Acad Sci U S A (1990) 87:4125–9. 10.1073/pnas.87.11.4125 2161533 PMC54060

[26] Bell MP Pavelko KD. Enhancing the tumor selectivity of a picornavirus virotherapy promotes tumor regression and the accumulation of infiltrating CD8+ T cells. Mol Cancer Ther (2016) 15:523–30. 10.1158/1535-7163.MCT-15-0459 26823492 PMC4783249

[27] Pavelko KD Bell MP Karyampudi L Hansen MJ Allen KS Knutson KL The epitope integration site for vaccine antigens determines virus control while maintaining efficacy in an engineered cancer vaccine. Mol Ther (2013) 21:1087–95. 10.1038/mt.2013.52 23568262 PMC3666639

[28] Rodriguez M Leibowitz JL Powell HC Lampert PW. Neonatal infection with the Daniels strain of Theiler’s murine encephalomyelitis virus. Lab Invest (1983) 49:672–9. 6361374

[29] Wonderlich J Shearer G Livingstone A Brooks A. Induction and measurement of cytotoxic T lymphocyte activity. Curr Protoc Immunol (2006) Chapter 3:Unit 3.11. 10.1002/0471142735.im0311s72 18432971

[30] Pulko V Harris KJ Liu X Gibbons RM Harrington SM Krco CJ B7-H1 expressed by activated CD8 T cells is essential for their survival. J Immunol (2011) 187:5606–14. 10.4049/jimmunol.1003976 22025548 PMC3221917

[31] Rodriguez M David CS. Demyelination induced by Theiler’s virus: influence of the H-2 haplotype. J Immunol (1985) 135:2145–8. 2991380

[32] Myoung J Hou W Kang B Lyman MA Kang JA Kim BS. The immunodominant CD8+ T cell epitope region of Theiler’s virus in resistant C57BL/6 mice is critical for anti-viral immune responses, viral persistence, and binding to the host cells. Virology (2007) 360:159–71. 10.1016/j.virol.2006.09.045 17095033 PMC1857342

[33] Wakim LM Woodward-Davis A Liu R Hu Y Villadangos J Smyth G The molecular signature of tissue resident memory CD8 T cells isolated from the brain. J Immunol (2012) 189:3462–71. 10.4049/jimmunol.1201305 22922816 PMC3884813

[34] Gibbons RM Liu X Pulko V Harrington SM Krco CJ Kwon ED B7-H1 limits the entry of effector CD8(+) T cells to the memory pool by upregulating Bim. Oncoimmunology (2012) 1:1061–73. 10.4161/onci.20850 23170254 PMC3494620

[35] Nitayaphan S Toth MM Roos RP. Neutralizing monoclonal antibodies to Theiler’s murine encephalomyelitis viruses. J Virol (1985) 53:651–7. 2578578 10.1128/jvi.53.2.651-657.1985 PMC254681

[36] Prasad S Hu S Sheng WS Chauhan P Singh A Lokensgard JR. The PD-1: PD-L1 pathway promotes development of brain-resident memory T cells following acute viral encephalitis. J Neuroinflammation (2017) 14:82. 10.1186/s12974-017-0860-3 28407741 PMC5390367

[37] Dai H Wan N Zhang S Moore Y Wan F Dai Z. Cutting edge: programmed death-1 defines CD8+CD122+ T cells as regulatory versus memory T cells. J Immunol (2010) 185:803–7. 10.4049/jimmunol.1000661 20548035

[38] Prabhu N Ho AW Wong KH Hutchinson PE Chua YL Kandasamy M Gamma interferon regulates contraction of the influenza virus-specific CD8 T cell response and limits the size of the memory population. J Virol (2013) 87:12510–22. 10.1128/JVI.01776-13 24027334 PMC3838152

[39] Liu Y Janeway CA Jr. Interferon gamma plays a critical role in induced cell death of effector T cell: a possible third mechanism of self-tolerance. J Exp Med (1990) 172:1735–9. 10.1084/jem.172.6.1735 2147950 PMC2188763

[40] Refaeli Y Van Parijs L Alexander SI Abbas AK. Interferon gamma is required for activation-induced death of T lymphocytes. J Exp Med (2002) 196:999–1005. 10.1084/jem.20020666 12370261 PMC2194022

[41] Intlekofer AM Takemoto N Kao C Banerjee A Schambach F Northrop JK Requirement for T-bet in the aberrant differentiation of unhelped memory CD8+ T cells. J Exp Med (2007) 204:2015–21. 10.1084/jem.20070841 17698591 PMC2118697

[42] Hawke S Stevenson PG Freeman S Bangham CR. Long-term persistence of activated cytotoxic T lymphocytes after viral infection of the central nervous system. J Exp Med (1998) 187:1575–82. 10.1084/jem.187.10.1575 9584136 PMC2212297

[43] Bergmann CC Altman JD Hinton D Stohlman SA. Inverted immunodominance and impaired cytolytic function of CD8+ T cells during viral persistence in the central nervous system. J Immunol (1999) 163:3379–87. 10477608

[44] Blackburn SD Crawford A Shin H Polley A Freeman GJ Wherry EJ. Tissue-specific differences in PD-1 and PD-L1 expression during chronic viral infection: implications for CD8 T-cell exhaustion. J Virol (2010) 84:2078–89. 10.1128/JVI.01579-09 19955307 PMC2812396

[45] Vargas-Inchaustegui DA Xiao P Hogg AE Demberg T McKinnon K Venzon D Immune targeting of PD-1(hi) expressing cells during and after antiretroviral therapy in SIV-infected rhesus macaques. Virology (2013) 447:274–84. 10.1016/j.virol.2013.09.015 24210124 PMC3869407

[46] Barber DL Wherry EJ Masopust D Zhu B Allison JP Sharpe AH Restoring function in exhausted CD8 T cells during chronic viral infection. Nature (2006) 439:682–7. 10.1038/nature04444 16382236

[47] Boni C Fisicaro P Valdatta C Amadei B Di Vincenzo P Giuberti T Characterization of hepatitis B virus (HBV)-specific T-cell dysfunction in chronic HBV infection. J Virol (2007) 81:4215–25. 10.1128/JVI.02844-06 17287266 PMC1866111

[48] Odorizzi PM Pauken KE Paley MA Sharpe A Wherry EJ. Genetic absence of PD-1 promotes accumulation of terminally differentiated exhausted CD8+ T cells. J Exp Med (2015) 212:1125–37. 10.1084/jem.20142237 26034050 PMC4493417

[49] Zozulya AL Ortler S Fabry Z Sandor M Wiendl H. The level of B7 homologue 1 expression on brain DC is decisive for CD8 Treg cell recruitment into the CNS during EAE. Eur J Immunol (2009) 39:1536–43. 10.1002/eji.200839165 19424967 PMC2889907

[50] Phares TW Ramakrishna C Parra GI Epstein A Chen L Atkinson R Target-dependent B7-H1 regulation contributes to clearance of central nervous system infection and dampens morbidity. J Immunol (2009) 182:5430–8. 10.4049/jimmunol.0803557 19380790 PMC2909606

[51] Salama AD Chitnis T Imitola J Ansari MJ Akiba H Tushima F Critical role of the programmed death-1 (PD-1) pathway in regulation of experimental autoimmune encephalomyelitis. J Exp Med (2003) 198:71–8. 10.1084/jem.20022119 12847138 PMC2196082

[52] Bodhankar S Chen Y Vandenbark AA Murphy SJ Offner H. PD-L1 enhances CNS inflammation and infarct volume following experimental stroke in mice in opposition to PD-1. J Neuroinflammation (2013) 10:111. 10.1186/1742-2094-10-111 24015822 PMC3846120

[53] Ritzel RM Crapser J Patel AR Verma R Grenier JM Chauhan A Age-associated resident memory CD8 T cells in the central nervous system are primed to potentiate inflammation after ischemic brain injury. J Immunol (2016) 196:3318–30. 10.4049/jimmunol.1502021 26962232 PMC4868658

[54] Gemechu JM Bentivoglio M. T cell recruitment in the brain during normal aging. Front Cell Neurosci (2012) 6:38. 10.3389/fncel.2012.00038 23049498 PMC3446775

[55] Barouch DH Deeks SG. Immunologic strategies for HIV-1 remission and eradication. Science (2014) 345:169–74. 10.1126/science.1255512 25013067 PMC4096716

[56] Zou W Wolchok JD Chen L PD-L1 (B7-H1) and PD-1 pathway blockade for cancer therapy: mechanisms, response biomarkers, and combinations. Sci Transl Med (2016) 8:328rv324 10.1126/scitranslmed.aad7118 PMC4859220 26936508

[57] Iwasaki A. Immune regulation of antibody access to neuronal tissues. Trends Mol Med (2017) 23:227–45. 10.1016/j.molmed.2017.01.004 28185790 PMC5626569

