# Supplementary figures and images for: B7-H1 Influences the Accumulation of Virus-Specific Tissue Resident Memory T Cells in the Central Nervous System
Source: Front Immunol. 2017 Nov 9;8:1532. doi: 10.3389/fimmu.2017.01532 (PMC5684101; doi:10.3389/fimmu.2017.01532)

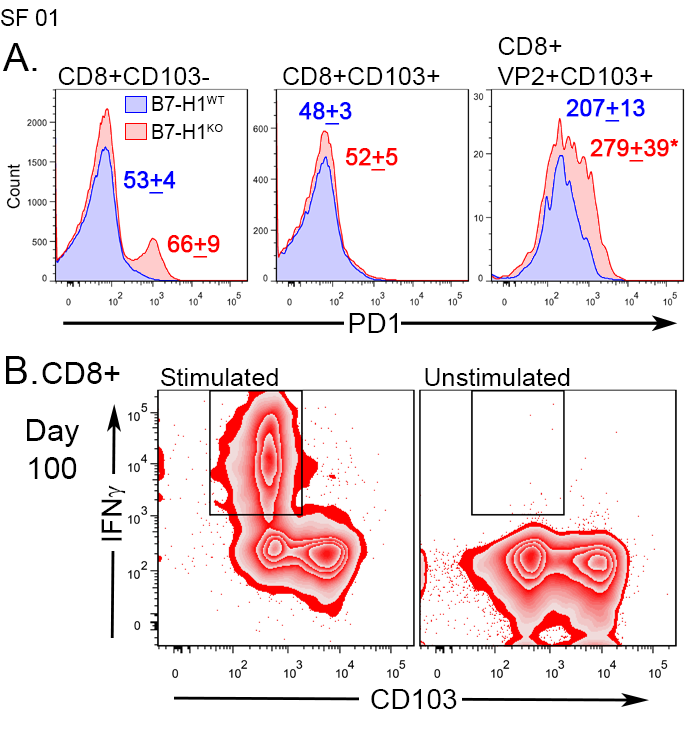

Supplement: Supplementary file 1 [file Image_1.TIF]

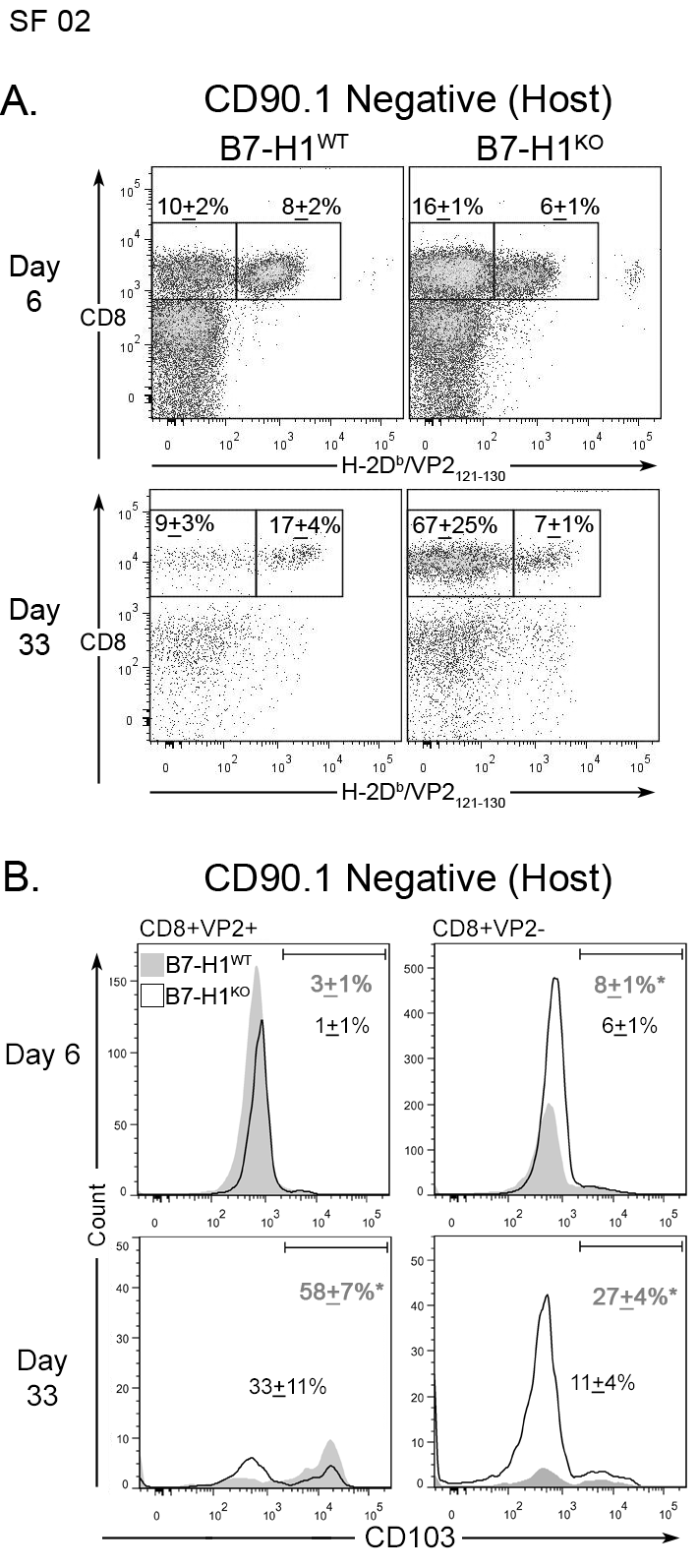

Supplement: Supplementary file 2 [file Image_2.TIF]
