# Supplementary material for: B7-H1 Influences the Accumulation of Virus-Specific Tissue Resident Memory T Cells in the Central Nervous System
Source: Front Immunol. 2017 Nov 9;8:1532. doi: 10.3389/fimmu.2017.01532 (PMC5684101; doi:10.3389/fimmu.2017.01532)
Supplement: Supplementary file 3 [file Presentation_1.PDF]

Supplementary Figure Legends

Figure S1. Expression of PD-1 and IFNγ in CD8+ splenocytes derived from mice intracranially infected with TMEV-OVA8 for >98 days. (A) PD-1 expression levels in CD103- CD8+, CD103+ CD8+ and VP2+ CD103+ CD8+ T-cell populations recovered from the spleen of B7-H1^{WT} and B7-H1^{KO} mice (Mean fluorescence intensity $\pm$STD; n=3 mice per group). * Significant by t-test $p<0.05$; B7-H1^{WT} versus B7-H1^{KO}. (B) IFNγ secretion relative to CD103 expression in CD8+ T-cells recovered from the spleen of B7-H1^{KO} mice infected with TMEV-OVA8 for 100 days. Pooled cells (n=6) were stimulated with PMA/Ionomycin overnight and are compared to unstimulated cells from the same pool.

Figure S2. Effects of T-cell and host derived B7-H1 on the accumulation of endogenous CD8+ T-cells after transfer of wild-type CD90.1+ CD8+ OT-1. (A) CD45+ gated endogenous CD90.1 negative cells (B7-H1^{WT} and B7-H1^{KO}) were analyzed for CD8 and H-2D^{b}-VP2$_{121\text{-}130}$ specificity (Percent of total recovered CD90.1- cells $\pm$ STD). (B) CD103 expression from VP2+ and VP2- cell populations in (A) (% CD103^{+} $\pm$ STD; n=5 per group). * Significant by t-test $p<0.05$; B7-H1^{WT} versus B7-H1^{KO}.
